# Troponin, ECG Changes, and Blood Pressure in Hypertensive Emergencies

**DOI:** 10.7759/cureus.114286

**Published:** 2026-08-10

**Authors:** Siddharth Singh, Abhay Kumar, Brajesh K Yadav, Santosh K Nayan, Rajbahadur Singh, Santosh Kumar, Anand Dev

**Affiliations:** 1 Emergency Medicine, Indira Gandhi Institute of Medical Sciences, Patna, IND; 2 Intensive Care Medicine, Indira Gandhi Institute of Medical Sciences, Patna, IND

**Keywords:** blood pressure, ecg, hypertensive emergencies, myocardial injury, troponin

## Abstract

Introduction and aim: Hypertensive emergencies, defined as an abrupt and severe elevation in blood pressure with acute target-organ damage, represent a critical condition with substantial morbidity and mortality. Cardiac involvement, particularly myocardial injury, is common but often under-recognized in acute settings. Measurement of cardiac troponin and electrocardiographic (ECG) assessment is essential for identifying myocardial damage, guiding early intervention, and prognostic evaluation in patients with hypertensive emergencies. This study aimed to evaluate the relationship between cardiac troponin levels, ECG changes, and blood pressure severity in predicting outcomes, including mortality, among patients with hypertensive emergencies admitted to the emergency department.

Methods: A prospective observational study at a tertiary care center in Eastern India enrolled 140 patients with systolic blood pressure (SBP) ≥180 mmHg and diastolic blood pressure (DBP) ≥110 mmHg with target-organ damage. Exclusion criteria included trauma, malignancy, and infection. All underwent detailed clinical assessment, ECG within 30 min, and high-sensitivity troponin I testing. Analyses included chi-square, Kruskal-Wallis, and multivariate logistic regression; p<0.05 was considered significant.

Results: Mean age was 50.21±16.52 years; 76 (54.29%) patients were male, and 84 (60%) patients were aged >45 years. Common symptoms were giddiness in 73 (52.14%) patients, nausea/vomiting in 67 (47.86%) patients, and headache in 55 (39.29%) patients. Altered mental status was noted in 38 (27.14%) patients and breathlessness in 39 (27.86%) patients and were strongly associated with mortality (p<0.001 and p=0.002). Elevated troponin (>150 pg/mL) occurred in 37 (26.43%) cases and independently predicted mortality (OR=4.79). ECG abnormalities occurred in 88 (62.86%) cases, mainly left ventricular hypertrophy in 97 (69.28%) cases. Mortality was noted in 25 (17.86%) cases, with 17 (12.14%) after 72 h.

Conclusion: Elevated troponin, ECG abnormalities, altered mental status, and breathlessness are strong mortality predictors in hypertensive emergencies, warranting early detection for improved outcomes.

## Introduction

Hypertensive emergencies are acute, life-threatening conditions characterized by a sudden rise in blood pressure with evidence of end-organ damage. Immediate intervention is critical to prevent complications such as pulmonary edema, myocardial ischemia, acute renal failure, stroke, and aortic dissection. Most cases occur in patients with chronic hypertension and are often triggered by medication nonadherence or sympathomimetic use [1].

The hypertensive crisis spectrum includes hypertensive urgency and hypertensive emergency, encompassing conditions such as malignant hypertension, which is diagnosed by severe blood pressure (BP) elevations, often >200/120 mmHg, with advanced retinopathy, including papilledema, hemorrhages, and cotton-wool spots. Additional forms include hypertensive encephalopathy, acute coronary syndrome, and stroke [2,3].

Pathophysiologically, acute BP surges cause vascular shear stress and contribute to microvascular damage, endothelial dysfunction, and reduced coronary perfusion, ultimately resulting in myocardial injury. Myocardial necrosis, in turn, triggers the release of cardiac troponins, a key biomarker of myocardial injury [4]. Concurrent structural cardiac changes may manifest as ECG abnormalities, such as ST-segment changes, T-wave inversions, and arrhythmias, serving as both diagnostic and prognostic markers of adverse outcomes [3].

Hypertensive crises can cause cerebral autoregulatory failure, resulting in high rates of hemorrhagic stroke, cerebral ischemia, and encephalopathy, which may explain the significant association between altered mental status and mortality [5]. Respiratory compromise in hypertensive emergencies can lead to pulmonary congestion and respiratory distress, which may indicate acute heart failure or neurogenic pulmonary edema, both of which have been linked to a poor prognosis. The significant association between breathlessness and mortality highlights the need for early recognition of respiratory distress and prompt intervention with diuretics, vasodilators, and respiratory support [6].

In recent years, the role of cardiac troponins and ECG changes in hypertensive emergencies has gained increasing attention. Cardiac troponins are highly sensitive and specific markers of myocardial injury and are routinely used in the diagnosis of acute coronary syndromes. In the context of hypertensive emergencies, elevated troponin levels suggest myocardial damage not necessarily due to coronary obstruction but rather to hemodynamic overload and vascular stress [3]. However, interpreting these values can be challenging because of confounding factors such as renal dysfunction, sepsis, and chronic myocardial strain.

## Materials and methods

This prospective observational cohort study was conducted over a period of 12 months, from July 1, 2024 to June 30, 2025, at a tertiary care center in Eastern India. The primary objective of this study was to evaluate the prognostic significance of cardiac troponin levels, ECG abnormalities, and blood pressure severity in predicting in-hospital mortality among patients presenting with hypertensive emergencies. The secondary objectives were to assess the association of these parameters with hypertension-mediated end-organ involvement, including cardiovascular and neurological complications, and to describe the clinical profile of patients with hypertensive emergencies.

The study included patients presenting with hypertensive emergencies who were admitted within 24 h of symptom onset. Eligible participants were adults aged 18-60 years with confirmed hypertensive emergencies, including cases of hemorrhagic stroke, hypertension-mediated organ damage (HMOD), and pregnant women with preeclampsia or eclampsia. Patients younger than 18 years or older than 60 years, those with incomplete medical records, trauma, malignancy, immunodeficiency, chronic kidney disease or AIDS, and individuals or legal guardians who declined consent were excluded from the study. Patients between the ages of 18 and 60 years were included to minimize confounding from advanced age-related comorbidities and frailty, which may independently influence cardiac biomarkers and clinical outcomes.

Patients with hypertensive crisis reportedly account for 3.2% of emergency department (ED) visits, and approximately 1-2% of hypertensive patients experience a hypertensive crisis during their lifetime [7]. Assuming that the incidence among patients presenting to the ED with hypertensive crisis increased to 10%, a sample size of 140 patients would be required to estimate the expected proportion with an absolute precision of 5% and a 95% confidence level.

All emergency admissions were screened, and patients meeting the inclusion criteria were recruited after obtaining informed consent. Demographic characteristics, comorbidities (diabetes mellitus, hypertension, coronary artery disease, and chronic kidney disease), lifestyle factors (smoking and alcohol use), clinical parameters (including Glasgow Coma Scale score, vital signs, SpO₂, ECG findings, and arterial blood gas analysis), and laboratory investigations, with particular emphasis on cardiac troponin levels, were documented at admission. High-sensitivity cardiac troponin I (hs-TnI) was assessed by measuring serum troponin levels by chemiluminescent microparticle immunoassay method on Abbott Architect i2000 (Irving, TX: Abbott Laboratories, Inc.), with reference range less than 15.6 pg/mL. Serial measurements of hs-TnI were performed at admission and when clinically indicated to differentiate acute myocardial infarction from myocardial injury related to hypertensive emergencies. The 12-lead ECGs obtained at admission were interpreted by treating physicians and classified according to predefined abnormalities, including left ventricular hypertrophy, ST-segment changes, T-wave abnormalities, and arrhythmias. Contrast-enhanced computed tomography (CECT) of the brain was performed when clinically indicated to identify acute cerebrovascular events.

Data analysis was performed using SPSS version 27.0 (Armonk, NY: IBM Corp.). Continuous variables were expressed as mean±standard deviation (SD) and analyzed using analysis of variance (ANOVA) for comparisons across multiple groups. Tukey’s post hoc test was performed to assess intergroup differences. Categorical variables were expressed as frequencies and percentages. The chi-square (χ²) test was used to evaluate associations between categorical predictors (e.g., altered mental status, breathlessness, ECG changes, and troponin levels) and mortality outcomes. The Kruskal-Wallis test was used for nonparametric comparisons of continuous variables, such as age, systolic blood pressure (SBP), diastolic blood pressure (DBP), and duration of hospital stay, across mortality groups. Effect size was reported using epsilon squared (ε²). Multivariate logistic regression analysis was performed to identify independent predictors of mortality, and odds ratios (ORs) with 95% confidence intervals (CIs) were calculated. A p<0.05 was considered statistically significant.

## Results

A total of 140 patients were included in the study, with a mean age of 50.21±16.52 years. Notably, 84 (60%) patients were aged 45 years or older. Males constituted a slight majority, accounting for 76 (54.29%) males, compared with 64 (45.71%) females.

The most frequently reported symptoms in patients were as follows: giddiness in 73 (52.14%) patients, followed closely by nausea/vomiting in 67 (47.86%) patients and headache in 55 (39.29%) patients. Notably, altered mental status was observed in 38 (27.14%) patients and breathlessness in 39 (27.86%) patients, both of which were later found to have a significant association with mortality. Less commonly reported symptoms in patients included seizures (nine, 6.43%), visual disturbances (nine, 6.43%), and epistaxis (two, 1.43%).

Regarding patient comorbidities, diabetes mellitus (58 patients, 41.43%) and systemic hypertension (39 patients, 27.86%) were the most prevalent. A history of alcohol consumption was noted in 56 (40%) patients and smoking in 38 (27.14%) patients. Cardiovascular and renal conditions, including coronary artery disease in 21 (15%) patients and chronic kidney disease in 23 (16.43%) patients, were present in a smaller proportion of patients.

Hypertensive crisis was a prominent feature in the study population, with a mean systolic blood pressure (SBP) of 215.68±26.27 mmHg and a mean diastolic blood pressure (DBP) of 127.22±23.12 mmHg at presentation. These extreme values reflect the severity of the hypertensive emergency.

ECG abnormalities were detected in 88 (62.86%) cases, with isolated left ventricular hypertrophy (LVH) being the most common finding in 61 (69.28%) cases. ST changes were observed in 32 (22.85%) patients, and abnormal T waves were observed in 46 (32.85%) patients, suggesting underlying cardiac distress. Arrhythmias were less frequent, with atrial arrhythmias in nine (6.42%) patients and ventricular arrhythmias in three (2.14%) patients. Elevated hs-TnI levels were found in a high proportion of patients, with 37 (26.43%) patients exceeding 150 pg/mL and an additional 33 (23.57%) patients falling between 101-150 pg/mL, with value decreases in short duration, which differentiates it from myocardial infarction (MI).

Contrast-enhanced computed tomography (CECT) brain was performed in 69 (49.29%) patients, revealing acute hemorrhagic stroke in 48 (34.28%) patients and acute infarction in 33 (23.57%) patients. The mean duration of hospital stay was 9.85±6.25 days, reflecting the prolonged management often required for severe hypertensive emergencies. Inpatient mortality was observed in 25 (17.86%) cases, with eight (5.71%) occurring within the first 72 h and the remaining 17 (12.14%) after 72 h. The majority of patients (115, 82.14%) survived their hospitalization.

On performing the Kruskal-Wallis test, there was a significant association between age and mortality (χ²=6.06, p=0.048, ε²=0.0436), indicating that older patients were at higher risk. Blood pressure at admission was also a crucial determinant, with SBP (χ²=8.58, p=0.014, ε²=0.0617) and DBP (χ²=10.37, p=0.006, ε²=0.0746) showing significant associations with mortality. Interestingly, the duration of hospital stay demonstrated the strongest association (χ²=28.99, p<0.001, ε²=0.2085).

The chi-square test further revealed that altered mental status (χ²=17.21, p<0.001) and breathlessness (χ²=12.24, p=0.002) were significantly associated with mortality. However, gender (p=0.902), headache (p=0.239), giddiness (p=0.990), and chest pain (p=0.154) did not show any significant correlation with mortality.

Multivariate logistic regression identified elevated troponin levels as the strongest predictor of mortality (OR=4.79), reinforcing the role of myocardial injury in adverse outcomes. Altered mental status (OR=3.44) and breathlessness (OR=1.85) also emerged as significant risk factors. Interestingly, a longer hospital stay was associated with a lower mortality risk (OR=0.59).

The multivariate analysis demonstrated a significant association between troponin levels, ECG changes, and mortality outcomes. A total of 17 (68.0%) patients with troponin levels exceeding 150 pg/mL exhibited the highest mortality rate, particularly when ECG changes were present. In contrast, those with troponin levels below 15 pg/mL and no ECG changes showed no deaths. The chi-square test yielded a statistically significant result (χ²=24.66, p<0.001), confirming a strong relationship between troponin levels, ECG changes, and mortality (Table 1).

**Table 1:** Serum troponin and ECG changes with mortality.

Troponin level (pg/mL)	No ECG changes, n	ECG changes, n	Mortality, %
<15	30	21	0
15-100	10	9	12
101-150	7	26	20
>150	5	32	68

The mean systolic blood pressure (SBP) at arrival was categorized by troponin levels, ECG changes, and mortality outcomes. Patients with troponin levels >150 pg/mL had the highest mean SBP (236.6 mmHg), especially among those who later died beyond 72 h. This trend was observed consistently across different troponin levels, with increasing SBP aligning with higher troponin values (Table 2).

**Table 2:** Troponin level with ECG changes, mortality, and mean SBP. SBP: systolic blood pressure

Troponin level (pg/mL)	ECG change	Survived, n	Death in 72 h, n	Death after 72 h, n	Mean SBP at arrival (mmHg)
<15	Normal	27	0	0	197.5
<15	Changed	24	0	0	197.5
15-100	Normal	8	1	2	205.6
15-100	Changed	8	0	0	205.6
101-150	Normal	17	1	3	226.1
101-150	Changed	11	0	1	226.1
>150	Normal	16	4	9	236.6
>150	Changed	4	2	2	236.6

ANOVA revealed a significant difference in mean SBP across mortality groups (F=6.61, p=0.0018), suggesting that SBP at arrival varies significantly depending on mortality outcomes. A total of 58 patients had multiple comorbidities; six of them died within 72 h, and 15 of them died after 72 h.

On Tukey’s post hoc analysis, a significant mean SBP difference was observed between survivors and those who died after 72 h (22.34 mmHg, p=0.003), further clarifying these differences. No significant differences were detected between survivors and those who died within 72 h or between early and late deaths.

## Discussion

Hypertensive emergencies remain a major cause of acute target-organ damage and significant in-hospital mortality. Male predominance, diabetes, chronic hypertension, smoking, and alcohol use highlight the importance of aggressive risk-factor modification. Neurological and respiratory manifestations emerged as strong predictors of poor outcomes. Elevated systolic and diastolic blood pressures were strongly associated with mortality. ECG abnormalities, elevated troponin levels, and cerebrovascular complications, especially hemorrhagic stroke, are independent prognostic markers associated with increased in-hospital mortality and likely reflect the severity of underlying target-organ damage.

In our study, 84 (60%) patients were over the age of 45 years, with a mean age of 50.21±16.52 years, reflecting the increased vulnerability of this demographic to severe hypertensive complications. This finding is clinically relevant because aging is associated with vascular stiffening due to collagen deposition, impaired baroreceptor sensitivity, and cumulative exposure to cardiovascular risk factors such as dyslipidemia and insulin resistance. These factors may predispose individuals to abrupt decompensations in blood pressure control and magnify the consequences of acute surges. Our finding is supported by the study by Mills et al., who noted that the global burden of hypertension is increasing, particularly among middle-aged and elderly populations in low- and middle-income countries [8]. This epidemiological shift highlights the urgent need for targeted preventive and interventional measures in these age groups, including more aggressive screening, earlier pharmacological intervention, and patient education initiatives focused on long-term blood pressure maintenance.

The male predominance observed in our study (76, 54.29%) underscores that men are more frequently affected by uncontrolled hypertension and its complications, likely because of lifestyle factors and delayed healthcare-seeking behavior. Contributory factors may include a higher prevalence of tobacco and alcohol use among men, occupational stress, cultural norms surrounding masculinity and health, and poorer adherence to antihypertensive therapy despite awareness. Similar patterns were documented by Nagendra et al., reinforcing the importance of gender-specific strategies in hypertension screening and control programs [9]. Such strategies could involve workplace-based screening initiatives, public health messaging tailored to male populations, early lifestyle-modification counseling, and the use of digital health tools to improve follow-up in populations that may otherwise disengage from care.

In our cohort, giddiness was observed in 73 (52.14%), nausea/vomiting in 67 (47.86%) cases, and headache in 55 (39.29%) cases, making them the most frequently reported symptoms. However, altered mental status (38, 27.14%) and breathlessness (39, 27.86%) showed a significant association with mortality. The presence of altered mental status may reflect acute hypertensive encephalopathy, cerebrovascular events, severe metabolic derangements, or even posterior reversible encephalopathy syndrome. In contrast, breathlessness may signal acute left ventricular failure, pulmonary edema, or concomitant respiratory pathology. This also reflects the observations of Kowalski et al. that nonspecific symptoms are common in hypertensive crises and aligns with the findings of Zampaglione et al., who identified these symptoms as indicators of acute end-organ involvement and poor prognosis [7,10]. These observations emphasize the need for emergency clinicians to maintain a high index of suspicion, even when patients present with seemingly vague complaints, as subtle neurological or respiratory signs may precede catastrophic deterioration.

Diabetes mellitus (58, 41.43%) and hypertension (39, 27.86%) were the most prevalent comorbidities in our study and were accompanied by high rates of alcohol use (56, 40%) and smoking (38, 27.14%). Diabetes accelerates atherosclerosis and microvascular damage, thereby increasing susceptibility to hypertensive end-organ injury through both macrovascular occlusion and endothelial dysfunction. Similarly, pre-existing hypertension can exacerbate the rapid rise in blood pressure during crises, accelerating the progression toward irreversible damage. These results parallel the findings of Oh et al., who linked these comorbidities to a greater risk of organ damage during blood pressure surges, and those of Nagao et al., who reported that lifestyle factors such as heavy alcohol consumption and chronic smoking amplify the risk of hypertensive decompensation [11,12]. Incorporating aggressive risk-factor modification, including dietary interventions, structured exercise programs, and smoking-cessation support, into routine care could potentially reduce the frequency and severity of such emergencies.

We recorded mean SBP and DBP at presentation of 215.68±26.27 mmHg and 127.22±23.12 mmHg, respectively, both of which were significantly associated with mortality. Sustained pressures at this level are capable of overwhelming autoregulatory mechanisms in critical vascular beds, leading to endothelial damage, increased permeability, fibrinoid necrosis, and activation of the coagulation cascade. This finding supports the report by Whelton et al. that SBP ≥180 mmHg increases the risk of stroke, myocardial infarction, and death, as well as findings from the Intensive Blood Pressure Reduction in Acute Cerebral Hemorrhage Trial (INTERACT2) demonstrating that early aggressive blood pressure reduction improves functional outcomes in intracerebral hemorrhage [13,14]. These data advocate immediate but carefully controlled blood pressure lowering in high-risk patients to avoid hypoperfusion injury, particularly in those with chronic hypertension who may have shifted autoregulatory thresholds.

Electrocardiographic abnormalities were observed in 88 (62.86%) patients, with left ventricular hypertrophy (LVH) being the most common finding (97, 69.32%), followed by T-wave abnormalities (46, 32.85%) and ST-segment changes (32, 22.85%). The predominance of LVH is consistent with chronic pressure overload resulting from longstanding hypertension, whereas T-wave and ST-segment changes may represent acute subendocardial ischemia or repolarization abnormalities secondary to myocardial strain. These results extend the work of Tadic et al., who identified LVH as a marker of chronic hypertension and a predictor of arrhythmia risk, and of de Alencar et al., who emphasized the prognostic role of ECG changes in acute myocardial stress [15,16]. The inclusion of routine ECG assessment in initial evaluation protocols is therefore warranted for prognostic insight and for identifying candidates who may require more intensive cardiac monitoring.

In our cohort, 37 (26.43%) patients had troponin levels >150 pg/mL, with a mortality rate of 68% (17) in this subgroup. This finding reinforces prior work by Kim et al. and Maheshwarappa and Rai, who found elevated troponin levels in hypertensive emergencies to be an independent predictor of mortality, even in the absence of acute coronary syndrome [17,18]. Elevated troponin I can be an independent prognostic marker associated with increased in-hospital mortality. Elevated troponin levels likely reflect a combination of myocardial oxygen supply demand mismatch, microvascular ischemia, catecholamine surge, and possible subclinical myocarditis triggered by severe hypertension.

Neuroimaging revealed acute hemorrhagic stroke in 48 (34.28%) patients and acute infarction in 33 (23.57%) patients who underwent imaging. The predominance of hemorrhagic events may be related to extreme blood pressure surges and structural cerebrovascular fragility within our population. These findings are consistent with those of Guiga et al., who identified cerebrovascular events as a common and often fatal manifestation of hypertensive emergencies, highlighting the need for prompt blood pressure control in neurologically symptomatic patients [19]. The high burden of neurovascular complications observed in our cohort supports routine neurological assessment, early neuroimaging, and rapid initiation of guideline-based stroke protocols in suspected cases.

Overall hospital mortality in our study was 25 (17.86%), with 17 (12.14%) deaths occurring beyond 72 h. A longer hospital stay was associated with a reduced risk of mortality (OR=0.59), suggesting a benefit from extended monitoring, careful titration of therapy, and management of delayed complications. Murty et al. and Panda et al. similarly observed that prolonged ICU or step-down care improves stabilization and recovery in high-risk hypertensive emergencies [20,21]. This observed association in our study is subject to survivor bias; hence, any causal interpretation cannot be ascertained.

Symptoms such as giddiness, chest pain, and headache did not correlate with mortality in our study, echoing the observations of Varounis et al. that not all presenting features possess prognostic value [22]. This finding reinforces that reliance solely on presenting symptoms can be misleading; instead, objective parameters, such as troponin levels, ECG changes, and altered mental status, should guide risk stratification and early intervention strategies.

Our statistical analysis demonstrated a strong association between these parameters and mortality (χ²=24.66, p<0.001), providing real-world support for the recommendation by Kim et al. to integrate troponin assessment, ECG findings, and mental status evaluation into early management strategies [17]. Such integration could refine triage, optimize resource allocation, and guide therapeutic intensity in emergency settings, ultimately improving survival rates.

The significant differences in mean SBP across mortality groups in our study, particularly between survivors and late deaths, mirror the findings of Quezada et al., who reported that low SBP increases mortality in acute pulmonary embolism, and those of Raphael et al., who described paradoxical effects of SBP in chronic heart failure [23,24]. These patterns highlight the complexity of blood pressure management, wherein both excessively high and excessively low values may be harmful, depending on the clinical context and the organ systems involved.

The high prevalence of hemorrhagic stroke (48, 34.28%) in our study is consistent with data from the Reduction of Atherothrombosis for Continued Health (REACH) registry and supports findings from the Systolic Hypertension in the Elderly Program (SHEP) trial suggesting that altered mental status predicts mortality [25,26]. Hemorrhagic stroke in hypertensive emergencies often presents abruptly with rapid neurological deterioration, and the associated risk of rebleeding or hematoma expansion necessitates immediate blood pressure control and neurosurgical evaluation when indicated.

Finally, the association between breathlessness and mortality observed in our study is consistent with observations from the Antihypertensive and Lipid-Lowering Treatment to Prevent Heart Attack Trial (ALLHAT), which linked dyspnea to higher rates of cardiac and renal failure. Likewise, our finding that longer hospital stays are protective aligns with the evidence presented by Suzuki et al., supporting proactive and prolonged management to reduce complications [27,28]. Together, these findings underscore the importance of early recognition of high-risk features, integration of multimodal monitoring, and sustained inpatient care to improve survival outcomes in patients with hypertensive emergencies.

This study has several limitations. First, it was conducted at a single tertiary care center with a relatively small sample size, which may limit the generalizability of the findings to other populations and healthcare settings. Second, the study population was restricted to patients aged 18-60 years; the results may not be applicable to older patients, who represent a substantial proportion of individuals presenting with hypertensive emergencies. Third, although multivariable logistic regression was performed, residual confounding factors cannot be excluded. Factors such as chronic kidney disease, pre-existing heart disease, occult coronary artery disease, and other conditions capable of influencing cardiac troponin levels may not have been fully accounted for. Fourth, the relatively small number of mortality events may have increased the risk of model overfitting, and the regression findings should therefore be interpreted with caution. Fifth, the primary outcome was all-cause in-hospital mortality, and cause-specific mortality was not systematically recorded, precluding subgroup analyses. In addition, the inverse association between hospital length of stay and mortality is susceptible to survivor (immortal time) bias. Finally, because of the observational design, the identified associations should not be interpreted as causal relationships. Larger prospective multicenter studies with longer follow-up are needed to validate these observations and establish their prognostic utility.

## Conclusions

Objective clinical parameters such as troponin elevation, ECG changes, and altered sensorium are more reliable prognostic indicators than nonspecific presenting symptoms in hypertensive emergencies. Integrating cardiac biomarkers, electrocardiographic assessment, and careful clinical evaluation may improve early risk stratification in these patients. However, given the observational single-center design, relatively small sample size, and potential residual confounding, these findings should be interpreted as associations rather than causal relationships.
